# Small for Gestational Age Preterm Neonates Exhibit Defective GH/IGF1 Signaling Pathway

**DOI:** 10.3389/fped.2021.711400

**Published:** 2021-08-10

**Authors:** Emmanuelle Motte-Signoret, Shivani Shankar-Aguilera, Sylvie Brailly-Tabard, Yohan Soreze, Valentina Dell Orto, Rafik Ben Ammar, Daniele De Luca, Pascal Boileau

**Affiliations:** ^1^Assistance Publique-Hôpitaux de Paris, Béclere Hospital, GH Paris Sud, Neonatal Intensive Care Unit, Clamart, France; ^2^Poissy St Germain Hospital, Neonatal Intensive Care Unit, Poissy, France; ^3^Paris-Saclay University, Université Versailles Saint Quentin, Institut National de Recherche pour l'Agriculture, l'Alimentation et l'Environnement, BREED, Jouy-en-Josas, France; ^4^Assistance Publique-Hôpitaux de Paris, Bicêtre Hospital, Molecular genetics Pharmacogenetics and Hormonology, Le Kremlin-Bicêtre, France; ^5^Institut National de la Santé et de la Recherche Médicale U1185, Le Kremlin-Bicêtre, France

**Keywords:** small for gestational age, preterm infants, fetal growth restriction, post-natal growth, idiopathic hyperglycemia, hormone resistance, metabolic syndrome, fetal programming 3

## Abstract

**Objective:** To investigate the impact of fetal growth restriction (FGR) on hormonal regulation of post-natal growth and glucose metabolism [via insulin and growth hormone (GH)/Insulin-like Growth factor 1 (IGF1) axis pathways] in small for gestational age (SGA) neonates.

**Methods:** We conducted a monocentric observational prospective comparative study on 73 singleton babies born with a weight inferior to 2,000 g. We analyzed auxological (weight, height and head circumference), and hormonal (GH, IGF1, and insulin plasma concentrations) data comparing SGA and appropriate for gestational age (AGA) neonates, between day 1 and 60.

**Results:** One third (23/73) of the neonates were SGA. Twenty-five percent (18/73) required insulin for idiopathic hyperglycemia of prematurity and were smaller in weight and head circumference at discharge. In the SGA group compared with the AGA group, GH plasma concentrations were higher at day 3 (70.1 vs. 38.0 mIU/L) and IGF1 plasma concentrations were higher at day 10 (29.0 vs. 18.7 ng/ml).

**Conclusions:** SGA neonates displayed resistance to GH and IGF1, concomitant to insulin resistance. This could partially explain the initial defective catch-up growth and, later in life, the higher prevalence of metabolic syndrome in this population.

## Introduction

Preterm birth is a major risk factor of neonatal morbidity and mortality which is worsened with fetal growth restriction (FGR) (1–3). In the 90's, Barker's work revealed that small for gestational age (SGA) neonates, defined as a birthweight below the 10th percentile for the gestational age, had an increased risk to develop metabolic syndrome when adults. This was the beginning of the concepts of fetal programming and the thrifty phenotype hypothesis (4, 5). Long term alterations in energetic metabolism affect these children with some of them exhibiting early insulin resistance (6). In addition, idiopathic hyperglycemia is frequent among extremely preterm or very low birth weight (VLBW) neonates (7, 8), and is usually explained by defects in glucose regulation with insulin resistance and lower insulin secretion (9, 10).

During fetal life and the first months after birth, growth is predominantly depending on nutrient intake and fetal insulin (11). The growth hormone (GH)/Insulin-like Growth factor 1 (IGF1) axis has a minor role in the regulation of growth during the first months of life, but is essential for glucose homeostasis (12). Maternal and placental GH are not known to impact fetal growth either (13). It is likely that insulin and GH/IGF1 endocrine systems share some of their intracellular signaling pathways and have synergistic anabolic and metabolic actions during the first weeks of extra-uterine life. Recently, murine models have emphasized the correlation between lack of catch-up growth in SGA rats and GH resistance (14) and the existence of cross-linked interactions between insulin and GH/IGF1 axis pathways (15).

Prematurity compounded with FGR expose the neonate to an higher risk of perinatal mortality (16), postnatal growth failure and ultimately metabolic syndrome in adult life (17). We hypothesized that FGR alters GH/IGF1 post-natal signalization with mechanisms similar to insulin signalization defects, and could thus affects post-natal growth of SGA neonates. We conducted a prospective study in 73 low birth-weight (LBW) neonates, describing auxological evolution and hormonal assay during their hospitalization in neonatal intensive care unit (NICU) in order to address our hypothesis.

## Methods

### Patients

This non-interventional comparative prospective cohort study was conducted in a single tertiary academic NICU between December 2015 and July 2016, with no modification of routine clinical care. It was approved by the local institutional review board (Paris Saclay University) and followed all the relevant local regulations (French Advisory Committee for the Protection of People, and CNIL n°2051804). Consent has been obtained from each patient's parent after full explanation of the purpose and nature of all procedures used.

All singleton, inborn neonates with birth weight <2,000 g admitted to the NICU were eligible for inclusion. Gestational age was determined by ultrasound assessment during the first trimester of pregnancy. Exclusion criteria were multiple pregnancies, uncertain gestational age at birth, maternal diabetes, maternal height lower than 150 cm, established chromosomal abnormality, genetic disease or skeletal dysplasia.

Patient management was standardized according to the ESPGHAN guidelines for enteral and parenteral nutrition (18). Glucose intakes were initially 6 to 8 g/kg/d, advanced by 1 to 2 g/kg/d and the goal was to reach 12–15 g/kg/d at the end of the first week. Glycaemia was measured on blood drops obtained by heel prick, and using point-of-care glucometer at the bedside. The administration of insulin for idiopathic neonatal hyperglycemia was initiated after three capillary glycaemia over 12 mmol/l, and introduced with intravenous continuous lispro insulin (initial posology 0.02 IU/kg/h secondarily adapted to capillary glycaemia sampled every 3 h).

### Data Collection

We collected clinical data [sex, gestational age, antenatal corticosteroids, etiology of prematurity, mode of delivery, insulin treatment, weight, height and head circumference (HC) at birth, around day 10, day 30 and day 60], in real time during hospitalization until discharge. Small and appropriate for gestational age (SGA and AGA) neonates were differentiated according to Fenton 2013 growth charts (19) taking for threshold the 10th percentile for weight and/or height. We defined extra-uterine growth retardation (EUGR) as growth values ≤ 10th percentile of intrauterine growth expectation based on estimated postmenstrual age in premature neonates at the time they are discharged from the hospital (20).

Plasma concentrations of IGF1, GH and insulin were measured between day 2 and 3, between day 8 and 10, and between day 25 and 35 using Sandwich immunometric assays (Immulite 2,000 XPi, Siemens Healthcare Diagnostics Erlangen, Germany for GH (detection thresholds: 3–1,000 mUI/L) and IGF1 (detection thresholds: 2–1,000 ng/mL) and Liaison, DiaSorin, Saluggia, Italy for Insulin, which does not crossmatch with lispro (detection thresholds: 0.87–500 μIU/mL)]. Blood was drawn through indwelling central venous lines or when not the case, by venipuncture at the same time of blood sampling for routine clinical care of the neonate. In case of venipuncture, non-pharmacological sedation (nursing 30% glucose solution) was given orally according to our clinical protocol, classically used in NICU without significantly modifying glycaemia.

We defined insulin resistance as the need for insulin treatment despite appropriate glucose intakes, and GH resistance as the association of elevated GH levels and extremely decreased IGF1 levels (below assay threshold).

### Statistical Analysis

Gaussian distributed data were expressed as mean +/- standard deviation. Differences in categorical variables were assessed by Chi2 or Fisher test, as appropriate. Continuous data were compared with Student test, correlation was tested with Pearson's coefficient.

We compared AGA vs. SGA groups, and patients requiring insulin therapy vs. the others (insulin and no insulin groups, respectively). The analysis was carried out with the statistical software GraphPad Prism 8.1. *P*-values <0.05 were considered statistically significant.

## Results

### Clinical Characteristics of the Study Population

**Figure 1:**
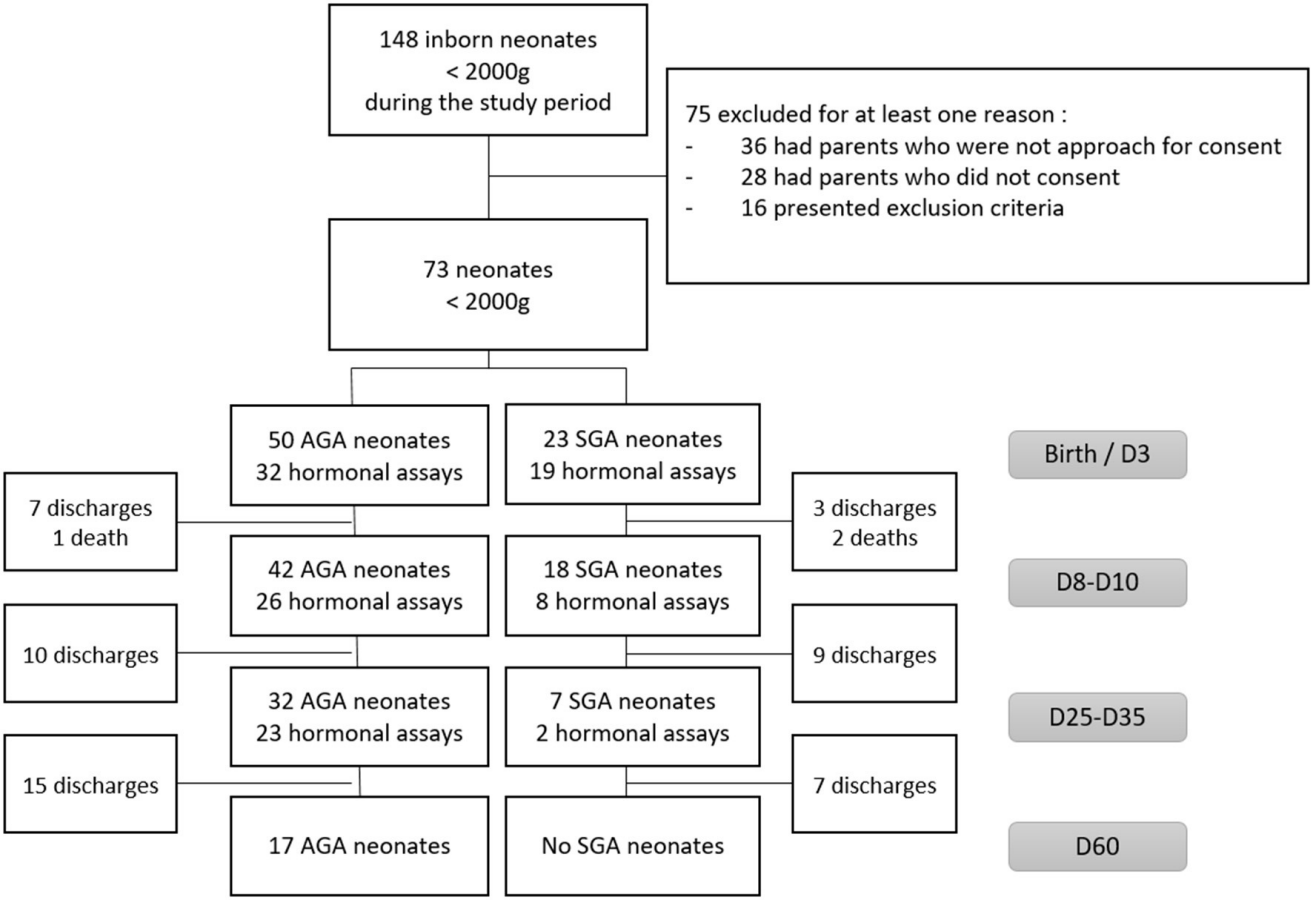
Flow chart of the study.

Seventy-three LBW neonates were included in our study, 22 (30%) were born before 28 weeks GA, 23 (32%) were SGA. Figure 1 describes the longitudinal evolution of the cohort: 60 babies (82%) were still hospitalized in our unit at day 10 (18 SGA), 39 (53%) at day 30 and only 17 (23%) at day 60. All SGA neonates had been discharged before day 60. Hormonal assays were performed in 51, 34, and 25 patients at day 3, 10, and 30, respectively.

Prematurity was medically induced in 49% (36/73) of all, and in 91% (21/23) of SGA neonates. Before 28 weeks of gestation, there was no preterm birth medically induced for fetal growth restriction (FGR). Conversely, preterm labor and preterm premature rupture of membranes rates were lower with a higher gestational age. Preterm birth was medically induced for FGR in 62% (14/23) of SGA neonates, whereas prematurity was spontaneous in 70% (35/50) of AGA neonates. Within the overall population, 26 neonates (36%) were extremely low birth weight (ELBW, <1,000 g), 22 (30%) and 55 (75%) were born before 28 and 32 weeks of gestation, respectively.

Birth auxological characteristics are described in Table 1. SGA neonates had a higher gestational age with a similar BW in comparison to AGA neonates. By definition, BW, BH and BHC in percentiles were significantly lower in SGA group. No difference was observed between the two groups for antenatal steroids. Eighteen (25%) neonates required insulin administration during their hospitalization, with a mean gestational age of 27.1 weeks and a mean BW of 881 g. Extremely preterm (<28 weeks) and ELBW infants had a significantly increased need for insulin treatment compared to the global study population (*p* = 0.0005 and *p* < 0.0001, respectively). There was no significant difference between AGA and SGA groups for need for insulin.

**Table 1:** Main characteristics of the study population.

	**All**	**<28 weeks GA**	**AGA**	**SGA**
N	73	22	50	23
GA (weeks)	29.8 ± 3.0	26.5 ± 1.2	28.6 ± 2.2	32.6 ± 2.5^***^
<28 wZeeks GA (%)	22 (30)	–	18 (36)	4 (17) ns
SGA (%)	23 (32)	4 (18)	–	–
BW (g)	1,153 ± 314	828 ± 156	1,118 ± 310	1,230 ± 315
BW (percentile)	33 ± 25	42 ± 21	45.6 ± 20.3	5.9 ± 4.6^***^
BH (cm)	37.0 ± 3.3	33.5 ± 2.0	36.6 ± 3.3	37.8 ± 3.2
BH (percentile)	34 ± 28	43 ± 20	47.5 ± 24	6.0 ± 6.3^***^
BHC (cm)	26.3 ± 3.6	23.5 ± 1.4	25.8 ± 2.6	27.2 ± 2.2^*^
BHC (percentile)	36 ± 27	40 ± 20	48.0 ± 24.5	10.4 ± 9.8^***^
Sex F/M	36/37	10/12	23/27	13/10
Antenatal steroids *n* (%)	57 (78)	18 (82)	39 (78)	18 (78) ns
Insulin therapy *n* (%)	18 (25)	12 (55)	15 (30)	3 (13) ns

*GA, gestational age; BW, BH, BHC, weight, height and head circumference at birth. Results expressed as mean ± standard deviation and percentage*.

*ns non significant*

* *p < 0.05*

*** *p < 0.001 for comparison between AGA vs. SGA groups*.

### Post-natal Growth

Thirty eight percent (15/39) of the babies exhibited EUGR at day 30 (8 within 32 AGA neonates (25%) vs. 100% of SGA neonates – *p* < 0.0001). Seventy one percent (12/17) of them had EUGR at day 60 and among them 90% were those who required insulin in the first days of life.

**Figure 2:**
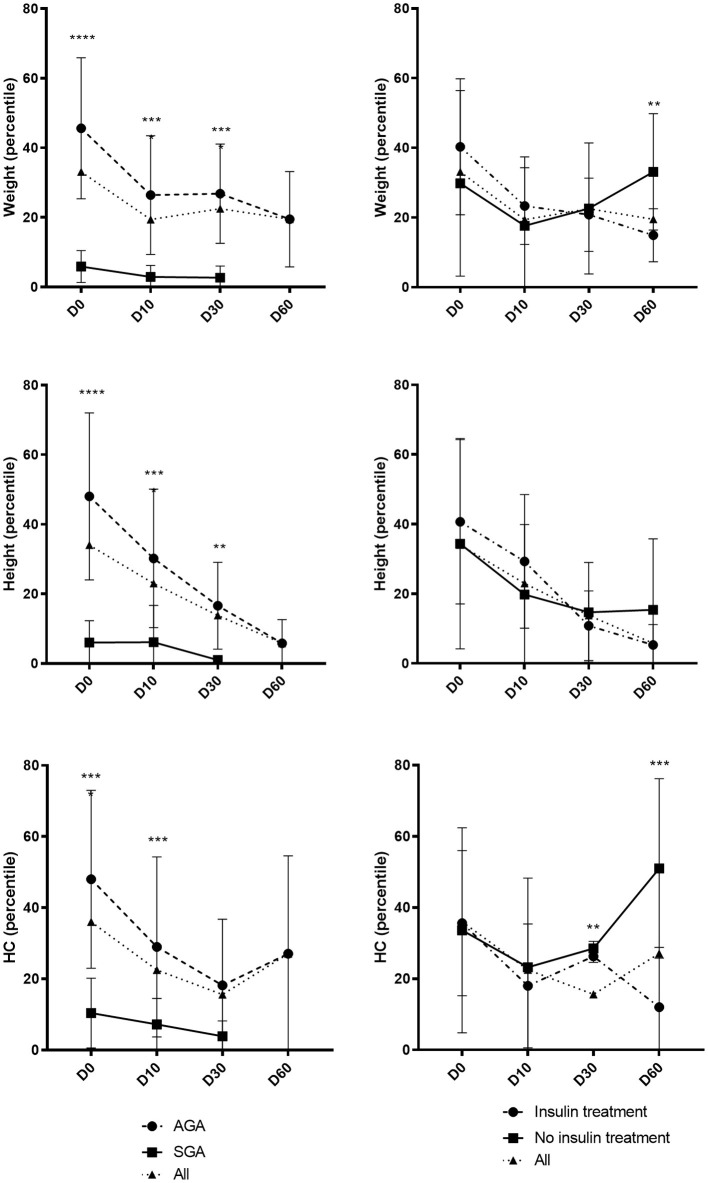
Evolution of growth parameters during the first 2 months of life. Day 0 (D0): all *n* = 73/AGA *n* = 50 vs. SGA *n* = 23/insulin *n* = 18 vs. no insulin *n* = 55. Day 10 (D10): all *n* = 60/AGA *n* = 42 vs. SGA *n* = 18/insulin *n* = 16 vs. no insulin *n* = 44. Day 30 (D30): all *n* = 39/AGA *n* = 32 vs. SGA *n* = 7/insulin *n* = 13 vs. no insulin *n* = 26. Day 60 (D60): all *n* = 17/AGA *n* = 17 vs. SGA *n* = 0/insulin *n* = 7 vs. no insulin *n* = 10. Results expressed in mean ± standard deviation. ^**^p < 0.01 ^***^p < 0.001 ^****^p < 0.0001.

The post-natal growth of the whole population and according to SGA/AGA and insulin/no insulin requirement status is illustrated in Figure 2. Weight and height in percentile of SGA neonates persisted to be significantly lower than the AGA neonates at birth, day 10 and day 30, while the HC was not significantly different between the two groups at day. Weight and HC of neonates requiring insulin were not significantly different than those without insulin at birth but were significantly lower at day 60.

### Hormonal Regulation

GH, IGF1 and insulin plasma concentrations are shown in Table 2 and Supplementary Figure 1. Fifty-one neonates had available measurements at day 3, 34 at day 10, and 25 at day 30 (respectively, 70, 47, and 34% of initial study population). We compared the results between AGA vs. SGA infants and between those who required insulin treatment vs. the others.

**Table 2:** mean plasma concentration of GH, IGF1 and insulin at day 3, 10 and 30 [mean ± standard deviation (min-max)].

		**GH (mIU/L)**	**IGF1 (ng/ml)**	**Insulin (mIU/L)**
Day 3	**All (** ***n*** **= 51)**	**50 ± 41 (5–208)**	**9 ± 8 (1–29)**	**27 ± 46 (1–307)**
	<28 weeks GA	42 ± 37 (11–110)	5.8 ± 6 (1–23)	34 ± 35 (2–131)
	AGA (*n* = 32)	38 ± 28 (6–110)	8 ± 8 (2–29)	25 ± 26 (1–131)
	SGA (*n* = 19)	70 ± 51 (5–208)^**^	10 ± 7 (1–25)	31 ± 74 (1–307)
	Insulin (*n =* 14)	44 ± 38 (5–110)	5 ± 7 (1–23)	56 ± 83 (5–208)
	No insulin (*n =* 47)	52 ± 42 (6–208)	10 ± 8 (1–29)	18 ± 20 (1–80)
Day 10	**All (** ***n =*** **34)**	**38 ± 25 (5–148)**	**21 ± 12 (1–40)**	**16 ± 16 (1–80)**
	<28 weeks GA	43 ± 44 (5–148)	11 ± 8 (1–25)	31 ± 25 (7–80)
	AGA (*n =* 26)	38 ± 28 (5–148)	19 ± 12 (1–40)	18 ± 19 (1–80)
	SGA (*n =* 8)	38 ± 10 (23–52)	29 ± 10 (13–39)^*^	11 ± 8 (1–23)
	Insulin (*n =* 10)	39 ± 42 (5–148)	16 ± 8 (1–25)	30 ± 23 (12–80)^*^
	No insulin (*n =* 24)	38 ± 16 (12–75)	24 ± 13 (1–40)	10 ± 9 (1–36)
Day 30	**All (** ***n =*** **25)**	**47 ± 29 (14–144)**	**26 ± 13 (1–48)**	**14 ± 14 (1–61)**
	<28 weeks GA	48 ± 26 (21–80)	18 ± 12 (1–48)	12 ± 6 (1–21)
	AGA (*n =* 23)	43 ± 20 (14–82)	25 ± 13 (1–48)	15 ± 14 (1–61)
	SGA (*n =* 2)	(35–144)	(37–40)	(2)
	Insulin (*n =* 8)	42 ± 27 (14–80)	23 ± 15 (13–48)	13 ± 5 (7–21)
	No insulin (*n =* 17)	50 ± 32 (17–144)	29 ± 11 (13–45)	15 ± 16 (1–61)

* *p < 0.05*

** *p < 0.01 for comparison between AGA vs. SGA or insulin vs. no insulin groups*.

GH plasma concentrations during the first month of life exhibited a mean range of 50, 38, and 47 mIU/l at day 3, day 10 and day 30 respectively. Forty two neonates (82%) exhibited GH levels to 20 mIU/L at day 3, 31 (91%) at day 10 and 23 (92%) at day 30. On the contrary, 4 (8%) neonates exhibited GH levels lower to 10 mIU/ml at day 3, 1 (3%) at day 10 and none at day 30. GH plasma concentrations were higher at day 3 in SGA (70.1 vs. 38.0 mIU/L; *p* = 0.004). We found no correlation between GH at birth and GA nor BW and a low correlation between GH and BW Fenton percentile (*R*^2^ = 0.09, *p* = 0.03).

IGF1 plasma concentrations exhibited a mean range of 7 ng/ml at birth, progressively increased during the first month (20 ng/ml at day 10 and 25 ng/ml at day 30) and were below the minimum threshold of the assay in 13 neonates (25%) at day 3, 2 (6%) at day 10 and 1 (4%) at day 30, regardless of weight, gestational age and weight for gestational age. At birth, IGF1 levels discretely correlated to GA (*R*^2^ = 0.08, *p* = 0.04) and BW (*R*^2^ = 0.14, *p* = 0.007) but not BW Fenton percentile. IGF1 plasma concentrations were higher at day 10 in SGA compared to AGA neonates (29 vs. 19 ng/mL; *p* < 0.001).

There was no significant difference of GH and IGF1 plasma concentrations during the first month of life between neonates needing insulin therapy and the others. Mean insulin levels were 15.5 mIU/ml at day 3 and decreased to 11.0 and 11.5 mIU/ml at day 10 and day 30. Low insulin levels (< 5 mIU/ml) were observed in 13 (25%), 7 (21%) and 3 (12%) neonates whereas 10 (20%), 2 (6%) and 1 (4%) neonates exhibited high insulin levels (> 40 mIU/ml) at day 3, day 10 and day 30, respectively. There were no significant differences in insulin plasma concentrations between AGA and SGA neonates. However, we found significantly higher insulin levels in babies requiring insulin treatment at day 10. There was no correlation between insulin plasma concentrations with either gestational age or birth weight.

At day 3, we found no correlation between GH and IGF1 plasma concentrations, for any gestational age or weight. However, 2 SGA neonates exhibited extremely high plasma concentrations of GH (208 and 171 mIU/L) with concomitant concentrations of IGF1 below the minimum threshold of the assay (<1 ng/ml). At day 30, GH plasma concentrations were inversely correlated to IGF1 levels (*R*^2^ = 0.38, *p* = 0.01) in AGA babies.

## Discussion

Glucose metabolism ontogenesis and fetal and neonatal growth are mainly regulated by insulin and GH/IGF1 axis pathways (21, 22). Prematurity and fetal growth restriction expose the neonate to growth failure and metabolic syndrome in childhood and when adults (17, 23–25). We have found that post-natal growth was altered in babies who required insulin for idiopathic hyperglycemia of prematurity and that SGA neonates were likely resistant to GH and IGF1. These results support the existence of cross-linked interactions between insulin and GH/IGF1 axis pathways, possibly contributing to a defect in growth and metabolic adaptation in this population of FGR babies.

There was several limitations in this prospective study conducted in 73 low birth weight preterm neonates, first prevalence of medically induced prematurity for FGR increases with the gestational age at birth due to obstetrical FGR management. Second, at day 60, an important number of patients has been lost to follow-up, including all the SGA neonates which had been discharged. Both limitations could have been source of significant bias in our study.

We first described post-natal growth and observed persistence in differences of auxological parameters between SGA and AGA groups during hospitalization. However, the differences were reduced progressively, mainly due to extra-uterine growth retardation (EUGR) in AGA neonates. We also found that preterm babies requiring insulin for idiopathic hyperglycemia, who had a higher birth weight percentile had lower weight and HC at day 60. Two others studies have found similar results: Scheurer et al. described that hyperglycemic preterm neonates were discharged with lower HC and had lower height, weight, and HC at four months post-discharge (26); Ramel and colleagues published that height, weight, and HC of hyperglycemic preterm neonates were lower at 36 months (27). These results are in accordance with our hypothesis of a resistance to the anabolic effect of insulin and concomitantly a poor response on growth of GH/IGF1 axis in this population.

In the second part, we analyzed plasma levels of GH, IGF1 and insulin. It has been reported that random GH values are clinically useful within the first month of life, and that serum IGF1 levels reflect spontaneous 24 h GH secretion (28, 29). GH plasma concentrations were elevated during the first month of life, as previously reported (28). A large proportion of the neonates exhibited spontaneous GH levels superior to 20 mIU/L (usual threshold used to define deficiency during GH stimulation test) while very few exhibited GH levels inferior to 10 mIU/ml. On the contrary, IGF1 plasma concentrations were very low at birth and progressively increased during the first month. They were frequently below the minimum threshold of the assay at birth. Overall, this suggests a resistance to GH action during the first month of life in this population.

At birth, there was no correlation between GH and BW or GA while IGF1 was discretely correlated to BW. However, BW percentile was correlated with GH levels, but not with IGF1 levels. GH and IGF1 plasma levels were also higher in SGA neonates, respectively, at day 3 and 10. GH and IGF1 plasma levels were not correlated before day 30. This suggests a higher level of resistance to GH and IGF1 in the first days of life in SGA neonates.

There are no precedent studies on GH plasma levels in this population but our results are in concordance with the few studies on IGF1 plasma levels in preterm babies (30). It has also been showed that IGF1 plasma concentration at birth was lower in SGA neonates (31), that mRNA and IGF1 protein were less expressed and translated in the placenta of SGA neonates (32). While IGF1 plasma levels were lower at birth they were found higher at 1 and 3 years in SGA children compared with AGA children (33). In addition, if SGA neonates do not exhibit catch-up growth before 3 years of life, IGF1 plasma levels remain lower at day 30 and 60 (30, 34).

Fetal growth relies on insulin and nutrient intake, and GH does not play a significant role in fetal and neonatal growth. Consistently, children with pan-hypopituitarism are not SGA and have no growth failure in the first months of life but frequently are hypoglycemic with low IGF1 levels (35). Conversely, children with defects in IGF1 signalization are SGA (36), and many polymorphisms in *IGF1* and *IGF1R* genes have been described associated with FGR (37, 38). This dissociation in the role of GH and IGF1 in fetal growth suggests that IGF1 secretion is partially independent of GH during this period. Fetal growth is largely due to insulin and nutrient intake [example of gestational diabetes mellitus (39)] but also to Insulin-like Growth Factor 2 (IGF2). This latter is central in the pathophysiology of Silver-Russel syndrome [SRS, low IGF2 and very SGA neonates (40)]. IGF1, IGF2 and insulin (and their respective receptors) share structural analogy, and their signalizations have cross-linked interactions (41). We suggest that SGA neonates, similarly to SRS children, might have a defect in the IGF system with compensatory increase in GH levels.

In our study, 22% of the neonates required insulin therapy for idiopathic hyperglycemia (45% among <28 weeks). All were born before 32 weeks and/or with a birth weight <1,500 g, concordant with previous findings (7). There was no correlation between insulin plasma levels and birth weight or gestational age. We observed a great variability in the plasma concentrations of insulin, especially among those who required insulin therapy. This is in accordance with the hypothesis that idiopathic hyperglycemia of the preterm neonate is related to both insulinopenia (due to abnormality in insulin biosynthesis during post-translational stages with pro-insulin accumulation) and insulin resistance (9, 10, 42). Nevertheless, considering that we analyzed neither the impact of nutritional intake nor the correlation with glycaemia during the insulin assay, these results should be taken with caution, even if all the neonates were managed with standardized procedures of enteral and parenteral nutrition according to ESPGHAN guidelines.

SGA neonates displayed a risk to develop metabolic syndrome (5, 23, 25) and SGA children have insulin resistance (43, 44). Some studies even correlate Homeostasis Model Assessment of Insulin Resistance (HOMA) index and higher IGF1 levels during childhood (45). Conversely, the absence of insulin resistance and better insulin secretion is correlated with a better catch-up growth (46, 47). Thus, post-natal growth and insulin sensitivity seem to be associated. Murine models have emphasized the correlation between lack of catch-up growth in SGA rats and GH resistance (14) and the existence of cross-linked interactions between insulin and GH/IGF1 axis pathways (15).

Overall, our results suggest GH and IGF1 resistance in addition to insulin resistance during the first weeks of life in SGA neonates.

## Data Availability Statement

The raw data supporting the conclusions of this article will be made available by the authors, without undue reservation.

## Ethics Statement

The studies involving human participants were reviewed and approved by CNIL n°2051804. Written informed consent from the participants' legal guardian/next of kin was not required to participate in this study in accordance with the national legislation and the institutional requirements.

## Author Contributions

EM-S, PB, and DD conceived and designed the study. EM-S, SS-A, YS, VD, and RB provided clinical data. SB-T performed the biochemical assays. EM-S and PB analyzed the data. EM-S drafted the article and SS-A, PB, and DD revised it critically for important intellectual content. All authors contributed to the article and approved the submitted version.

## Conflict of Interest

The authors declare that the research was conducted in the absence of any commercial or financial relationships that could be construed as a potential conflict of interest.

## Publisher's Note

All claims expressed in this article are solely those of the authors and do not necessarily represent those of their affiliated organizations, or those of the publisher, the editors and the reviewers. Any product that may be evaluated in this article, or claim that may be made by its manufacturer, is not guaranteed or endorsed by the publisher.

## Supplementary Material

Supplemental Figure 1 Correlation between GH/IGF1 plasma concentrations at birth (day 0 to 3) and gestational age (GA), birth weight (BW) and BW Fenton percentile (each point represents a single neonate, n=51).

## Abbreviations

FGR: fetal growth restriction
SGA: small for gestational age
AGA: appropriate for gestational age
GH: growth hormone
IGF1: insulin-like growth factor 1
IGF2: insulin-like growth factor 2
IU: international unit
NICU: neonatal intensive care unit
EUGR: extra-uterine growth retardation
HC: head circumference
VLBW: very low birth-weight
LBW: low birth-weight
SRS: silver-russel syndrome
HOMA: homeostasis model assessment of insulin resistance.
