# Supplementary figures and images for: Small for Gestational Age Preterm Neonates Exhibit Defective GH/IGF1 Signaling Pathway
Source: Front Pediatr. 2021 Aug 10;9:711400. doi: 10.3389/fped.2021.711400 (PMC8382944; doi:10.3389/fped.2021.711400)

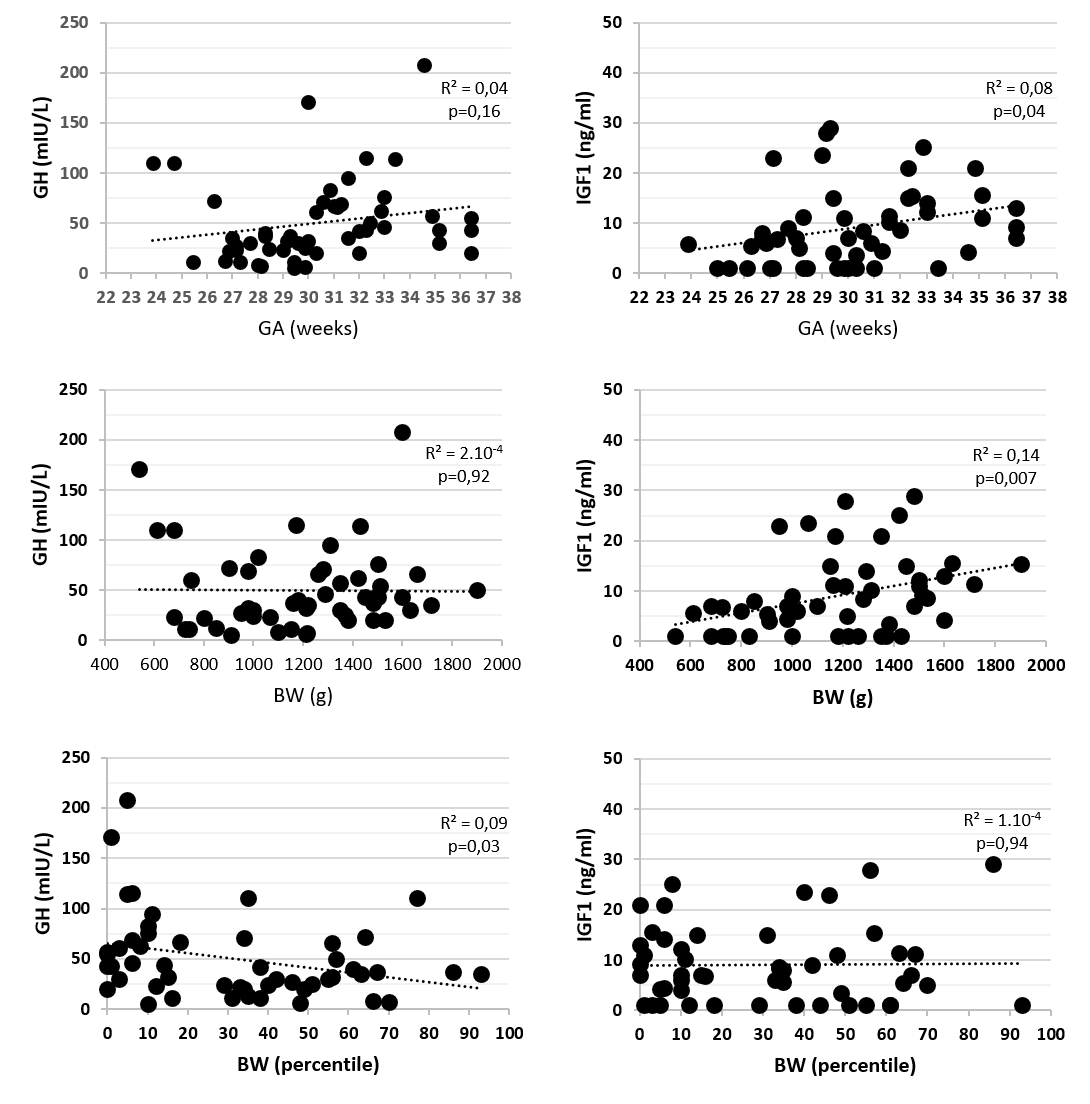

Supplement: Supplemental Figure 1 — Correlation between GH/IGF1 plasma concentrations at birth (day 0 to 3) and gestational age (GA), birth weight (BW) and BW Fenton percentile (each point represents a single neonate, n=51). [file Image_1.TIF]
